# Supplementary figures and images for: A rare CTSC mutation in Papillon-Lefèvre Syndrome results in abolished serine protease activity and reduced NET formation but otherwise normal neutrophil function
Source: PLoS One. 2021 Dec 21;16(12):e0261724. doi: 10.1371/journal.pone.0261724 (PMC8691626; doi:10.1371/journal.pone.0261724)

Raw Image - Immunoblot Fig 3

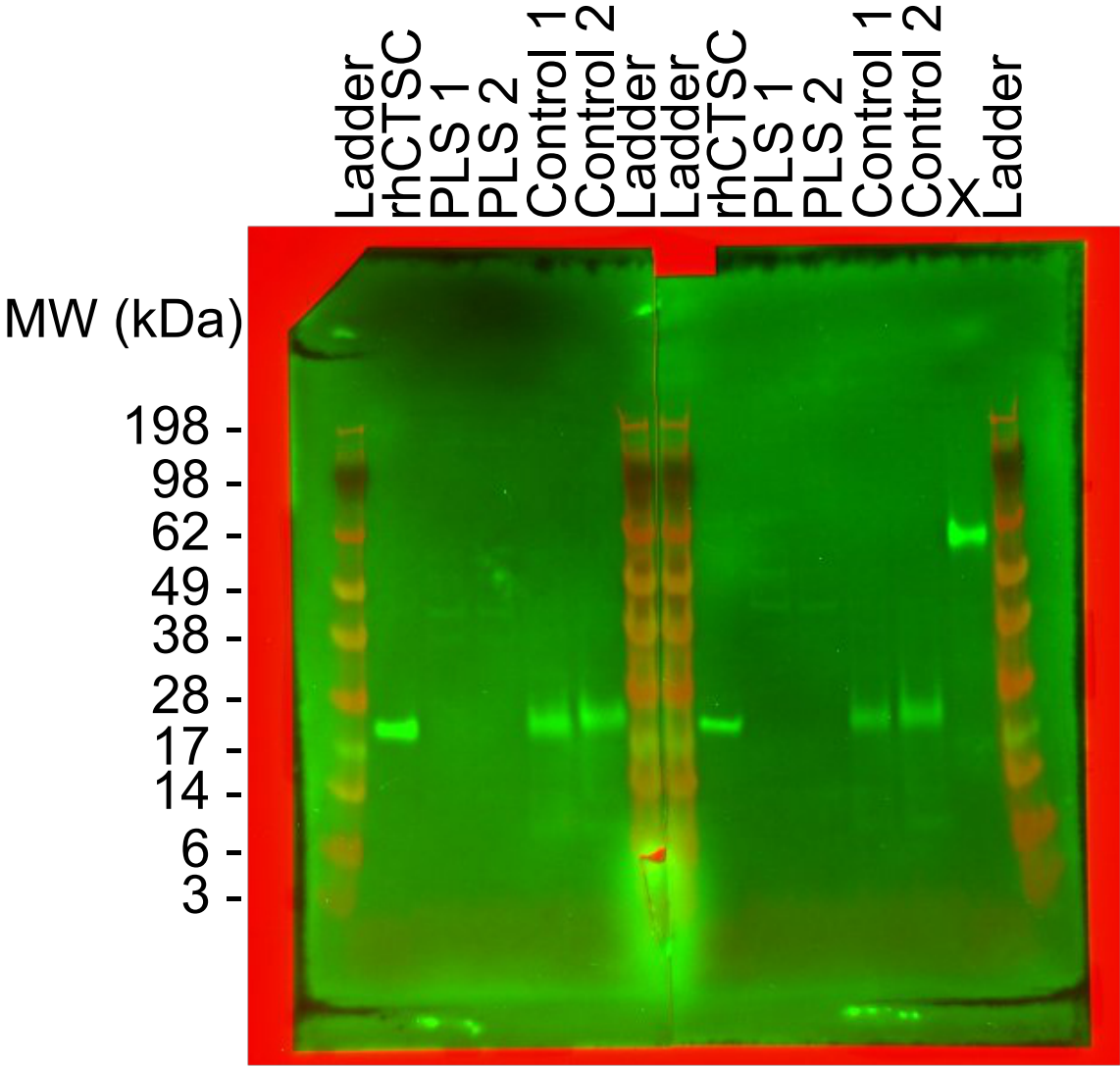

Supplement: S1 Raw images — (PDF) [file pone.0261724.s001.pdf]
